# Artificial intelligence in high-dose-rate brachytherapy treatment planning for cervical cancer: a review

**DOI:** 10.3389/fonc.2025.1507592

**Published:** 2025-01-27

**Authors:** Junyue Shi, Jun Chen, Gaokui He, Qinghe Peng

**Affiliations:** ^1^ Department of Nuclear Technology Application, China Institute of Atomic Energy, Beijing, China; ^2^ Department of Radiation Oncology, Foresea Life Insurance Guangzhou General Hospital, Guangzhou, China; ^3^ Department of Radiation Oncology, Sun Yat-sen University Cancer Center, State Key Laboratory of Oncology in South China, Collaborative Innovation Center for Cancer Medicine, Guangzhou, Guangdong, China

**Keywords:** artificial intelligence (AI), cervical cancer, high-dose-rate brachytherapy (HDR-BT), treatment planning, deep learning (DL)

## Abstract

Cervical cancer remains a significant global health concern, characterized by high morbidity and mortality rates. High-dose-rate brachytherapy (HDR-BT) is a critical component of cervical cancer treatment, requiring precise and efficient treatment planning. However, the process is labor-intensive, heavily reliant on operator expertise, and prone to variability due to factors such as applicator shifts and organ filling changes. Recent advancements in artificial intelligence (AI), particularly in medical image processing, offer significant potential for automating and standardizing treatment planning in HDR-BT. This review examines the progress and challenge of AI applications in HDR-BT treatment planning, focusing on automatic segmentation, applicator reconstruction, dose calculation, and plan optimization. By addressing current limitations and exploring future directions, this paper aims to guide the integration of AI into clinical practice, ultimately improving treatment accuracy, reducing preparation time, and enhancing patient outcomes.

## Introduction

Cervical cancer is a leading cause of cancer-related deaths among women worldwide, with high morbidity and mortality rates (1). High-dose-rate brachytherapy (HDR-BT) is an effective treatment method for cervical cancer, offering precise dose delivery to the tumor while sparing surrounding healthy tissues (2). However, HDR-BT treatment planning is a complex and time-sensitive process, requiring meticulous delineation of target areas (TVs) and organs at risk (OARs), accurate applicator reconstruction, and optimized dose calculations. The quality of the treatment plan is heavily dependent on the operator’s expertise, and delays between applicator insertion and treatment execution can compromise accuracy due to applicator shifts and changes in organ filling status (3, 4).

Recent advancements in artificial intelligence (AI) for medical image processing have introduced new opportunities to address these challenges. AI-driven methods, particularly deep learning (DL) models, have demonstrated significant potential in automating key steps of HDR-BT treatment planning, including segmentation, reconstruction, plan optimization and dose calculation. This review provides a comprehensive overview of the current state of AI applications in HDR-BT for cervical cancer, highlighting their potential to enhance clinical workflows, improve treatment accuracy, and reduce patient discomfort. Additionally, we discuss the challenges and limitations of AI integration in this field and propose future research directions to facilitate its clinical adoption.

## The application of AI in cervical cancer HDR-BT

### Automatic segmentation

UNet (5) is a popular convolutional neural network (CNN) architecture designed for effective image segmentation. Its unique U-shape structure makes it particularly well-suited for medical image segmentation, as it can handle features at multiple scales with excellent localization precision. The name “UNet” originates from its distinctive shape, comprising a contracting path to capture the global image context and a symmetric expanding path for precise localization. This design allows the network to efficiently extract relevant information from images while accurately classifying individual pixels. The application of UNet and its variants in the automatic segmentation of TVs and OARs during high-dose-rate brachytherapy (HDR-BT) for cervical cancer has been reported in several studies. For instance, Zhang et al. (6) developed a model named DSD-UNET based on the three-demensional (3D) UNet archeitecture (7) for the automatic contouring of the high-risk clinical target volume (HRCTV) and OARs. The model integrates an expansion convolution module in the middle part, utilizing convolutional kernels with different expansion rates to capture multi-scale features, thus achieving more accurate and stable image segmentation. Furthermore, the model introduces deep supervision in the expansion path, allowing it to generate the final output at different stages by integrating segmentation layers. The fusion of these multi-level feature map not only improves the reliability of segmentation, but also accelerates the training process. To evaluate model performance, the Dice similarity coefficient (DSC), Hausdorff distance (HD), and Jaccard index (JI) were used as evaluation metrics. The results showed that the DSD-UNET model performed significantly better than the 3D UNet model. Mohammadi et al. (8) designed the ResU-Net model by combining the characteristics of ResNet (9) and U-Net, which is used for the automatic segmentation of OARs. The model improves the efficiency and accuracy of the feature extraction process through long and short skip connections. Compared to manually defined standard OARs contours, the segmentation results show good consistency and outperform the classical U-Net model in all evaluation metrics. Jiang et al. utilized the RefineNet network model for automatic segmentation of HRCTV and OARs (10). RefineNet’s multi-path refinement network architecture excels at extracting raw contextual information directly from input images. Additionally, remote residual connections facilitate the learning of multi-scale and multi-level features, effectively enhancing higher-level convolutional layers with contextual details. This method had demonstrated high adaptability to variations in factors such as organ size, body shape, and age, successfully handling images with significant differences (11). The evaluation metrics, including DSC, HD, and overlap index (OI), indicated high consistency between automatic and manual delineation results for HRCTV and satisfactory performance for OARs like the bladder and rectum. However, the complex shapes and higher variability of the small intestine and sigmoid colon presented challenges for automatic delineation. Li et al. (12) used the adaptive ensemble network nnU-Net (13) for the automatic segmentation of HRCTV and OARs. They trained and integrated three different network architectures: 2D U-Net, 3D U-Net, and 3D Cascade U-Net. The results, evaluated quantitatively using metrics such as DSC, HD, ASD, and dosimetric parameters, showed that the 3D Cascade U-Net performed best in the segmentation of the bladder, rectum, and HRCTV. The dosimetric bias between automatic and manual delineation was within a clinically acceptable range. Cao et al. proposed a dual-path asymmetric weighting (DPAW) CNN-based segmentation method, aiming to leverage delineated HRCTV on MRI images before applicator insertion to guide automatic segmentation on post-insertion planning CT images (14). This method can overcome the issue of anatomical changes caused by the implantation of the applicator, vaginal filling, and the filling state of organs. Comparative analyses demonstrated that the dual-path CNN model outperformed its single-path counterpart in multiple dimensions, including diagnostic efficacy, segmentation accuracy, MRI data utilization, and model stability. Furthermore, the dual-path asymmetric weighting model surpassed the dual-path symmetric weighting model. To overcome the limitations of U-Net in extracting 3D information, Zhu et al. introduced the 2.5D model named SERes-u-net, which combines squeeze and excitation blocks with the ResNet network (15). By considering four adjacent slices across five channels as input, the model effectively manages complex and variable anatomical structures and spatial relationships, particularly in the intestine and sigmoid colon. Evaluations by clinical experts and quantitative metrics, including DSC and HD, confirmed the model’s strong performance in segmenting HRCTV, bladder, rectum, sigmoid colon, and intestine. While clinical assessments validated the model’s acceptability for critical organ segmentations, they also identified room for enhancement in sigmoid colon segmentation, which will be a future improvement area. Xue et al. developed the 3D Prompt-ResUNet module, integrating a prompt-based model with 3D nnU-Net for automatic segmentation (16). This module outperformed other state-of-the-art models, including nnU-Net and SAM-Med3D (17), in segmenting HRCTV, bladder, rectum, and sigmoid colon, as evidenced by superior DSC values. Yoganathan et al. designed three independent 2D networks for transverse, sagittal, and coronal planes, followed by constructing a 2.5D network, to automatically segment tumor target areas and OARs in MRI images (18). Evaluations using DSC, HD, and dosimetric parameters indicated significant advantage for the 2.5D network over the 2D network, with the InceptionResNetv2 model showing superior performance to ResNet50. Rodríguez et al. employed 3D nnU-Net for automatic tumor target segmentation and performed evaluations using metrics such as Dice, HD, and MSD, along with dosimetric assessments (19). Their research demonstrated the effectiveness of this automatic segmentation approach, with a median DSC of 0.73 and no significant differences in geometric and dosimetric performance compared to manual segmentation.

The self-attention mechanism in the Transformer architecture has the capability to capture long-range dependencies in images, effectively alleviating the limitations of convolutional neural networks (CNNs) in terms of their receptive fields. Zhu et al. developed a deep learning model called CoTr for automated tumor segmentation in cervical cancer magnetic resonance imaging (MRI) by integrating deep neural networks with the Vision Transformer (ViT) architecture. The CoTr model exhibited superior performance in segmentation tasks on contrast-enhanced T1-weighted (ceT1W) and T2-weighted (T2W) MR images, achieving average Dice similarity coefficients (DSCs) of 0.83 and 0.82, respectively. These results significantly outperformed other models that solely relied on CNN architectures, such as U-Net, Attention U-Net, and V-Net (20).

Moreover, recent advancements in multimodal image fusion segmentation algorithms have shown significant promise in improving segmentation accuracy for cervical cancer radiotherapy. Sun et al. proposed a novel segmentation network approach that employs synthetic images. Their method initially utilizes a generative network to transform CT images into T1W MR images. The synthesized MR images are then combined with the original CT images, allowing for automatic contouring of the CTV. This process mitigates the limitations of single-modality image information by harnessing the advantages of both CT and MRI. Specifically, it leverages the superior bone imaging capability of CT and the soft tissue contrast of MRI. Results demonstrate that this strategy effectively improves the accuracy of CTV delineation for clinical tumor targets in cervical cancer cases (21). **Table 1** summarizes the literature on AI-based automatic contouring in HDR-BT for cervical cancer.

**Table 1:** Literature summary of AI-based auto-contouring in high-dose-rate brachytherapy for cervical cancer.

Authors	Literature	Imaging	Network model	HRCTV		Bladder		Rectum		Sigmoid		Small intestine	
				DSC	HD	DSC	HD	DSC	HD	DSC	HD	DSC	HD
Zhang et al.	(6)	CT	DSD-UNET	0.82	/	0.87	/	0.83	/	/	/	/	/
Mohammadi et al.	(8)	CT	ResU-Net	/	/	0.96	4.1	0.97	2.0	0.92	3.2	/	/
Jiang et al.	(11)	CT	RefineNet	0.86	6.0	0.86	20.0	0.86	12.3	0.66	98.4	0.56	68.1
Li et al.	(12)	CT	nnU-Net	0.84	7.4	0.94	3.5	0.83	7.6	/	/	/	/
Gao et al.	(14)	CT	3D asymmetric CNN	0.79	5.5	/	/	/	/	/	/	/	/
Zhu et al.	(15)	CT	SERes-u-net	0.81	5.2	0.92	4.8	0.85	4.1	0.60	30	/	/
Xue et al.	(16)	CT	3D Prompt-ResUNet	0.92	8.5	0.93	3.1	0.87	3.5	0.76	7.5	/	/
Yoganathan et al.	(18)	MRI (T2W)	ResNet50	0.85	4.9	0.90	6.3	0.77	8.2	0.65	20.4	0.54	22.3
Rodríguez et al.	(19)	MRI (T2W)	3D nnU-Net	0.73	6.8	/	/	/	/	/	/	/	/
Zhu et al.	(20)	MRI (T1W + T2W)	CoTr	0.83	8.5	/	/	/	/	/	/	/	/
Sun et al.	(21)	CT + MRI (T1W)	GSN	0.95	0.9	/	/	/	/	/	/	/	/

DSC, dice similarity coefficient; HD, hausdorff distance; HRCTV, high-risk clinical target volume; CT, Computed Tomography; MRI, Magnetic Resonance.

Overall, the automatic contouring models currently used for HDR-BT perform well in the segmentation of the bladder and rectum, essentially achieving the precision of manual contouring. However, for the small intestine and sigmoid colon, due to their complex morphology and high variability, the performance of automatic contouring is still insufficient. Although the literature reports satisfactory results for the automatic contouring of tumor target areas, there is a lack of a gold standard and multicenter validation. Therefore, when applying AI technology to clinical HDR-BT for automatic contouring, careful verification of the structures outlined automatically and necessary manual adjustments are required. Additionally, most current research focuses on the automatic segmentation of CT images, while MRI guidance, as the gold standard for HDR-BT, benefits from its superior soft tissue contrast, which is conducive to more precise positioning and contouring of the HRCTV and OARs (22). Utilizing large standardized datasets for training is crucial for enhancing the generalization ability of automatic contouring models, learning deep features, optimizing network contouring accuracy, and improving robustness. The implementation of artificial intelligence technology to automatically segment the tumor target areas and OARs in cervical cancer HDR-BT can significantly reduce the time required for contouring and improve consistency. This not only reduces the waiting time for patients after brachytherapy applicator placement but also enhances the accuracy of treatment and patient comfort.

### Applicator reconstruction

Catheters reconstruction, the process of localizing the source path in the treatment planning image, is another crucial step in the HDR-BT treatment planning procedure. In this process, potential dwell positions are arranged on the digitized applicator channel, and dwell times are calculated to achieve dosimetric objectives. Given the unique steep dose gradient characteristic of HDR-BT, where radiation intensity rapidly decreases with distance from the source, the accuracy of applicator reconstruction significantly impacts the dosimetric outcomes of the treatment plan (23–25). Currently, applicator reconstruction in clinical practice is mostly performed manually by medical physicists, which is a subjective and time-consuming task. Therefore, fully automating reconstruction in 3D image-based HDR-BT treatment is particularly urgent, as it is vital for ensuring the accuracy and efficiency of the treatment plan. The applicator template library integrated into the treatment planning system serves as a powerful tool for clinical automatic digitization, significantly reducing uncertainties in the reconstruction process and improving efficiency. The library enables manual alignment based on virtual applicator models and predefined source paths, facilitating the digitization of the channel and ensuring its accurate location in the planning image. However, it is noteworthy that despite the significant advantages of the reconstruction method based on the template library in terms of efficiency and accuracy, it does not achieve full automation due to the requirement for manual alignment of the library models. Moreover, the applicability of this method is limited by the types of applicators available in the library. In the field of HDR-BT research, electromagnetic tracking technology, as an emerging approach, has recently been successfully applied to the source applicator digitization process. While this technology provides highly accurate digitization results, the additional hardware and complex procedures it requires pose potential barriers to its widespread adoption (26, 27). Dise et al. (28) combined the region growing algorithm with spline model of the catheters to successfully automate the source applicator digitization process, effectively avoiding incorrect digitization issues caused by overlapping sources and air bubble interference. However, it is worth noting that this method relies on manual seed point selection, and the reconstruction outcome is significantly influenced by the seed point location. In recent years, AI has been gradually introduced for the automatic reconstruction of applicators in HDR-BT. Deufel et al. (29) employed a U-Net model to automatically segment and reconstruct the Fletcher applicator used in cervical cancer HDR-BT from CT images. The results demonstrated excellent performance in terms of segmentation accuracy and reconstruction time, with an average DSC of 0.89, a HD of 1.66 mm, and a reconstruction time of only 17.12 seconds. The dose differences in HRCTV and OARs between automatic and manual reconstruction were within acceptable limits. Jung (30) first utilized a U-net network to segment interstitial needles in CT images, followed by clustering the segmented voxels into different needle groups and generating corresponding central trajectories. The results showed that the DSC between automatic and manual segmentation reached an average of 0.93, with a HD of approximately 0.71 mm for needle trajectories and an average deviation of less than 0.63 mm for needle tip positions, exhibiting clinically acceptable accuracy. Additionally, the entire digitization process could be completed within 5 minutes, highlighting the high efficiency of this method in clinical practice. Hu et al. (31) designed a 2D U-Net model to segment the contours of the applicators on the treatment planning CT images and employed a clustering algorithm to sort the channels. They utilized a polynomial curve-fitting method to obtain the central axis of the catheter and evaluated dose differences using a criterion of twice the maximum axis error (1 mm). The results indicated that the difference in the dose received by 90% of the volume of HRCTV between automatic and manual reconstruction was very small, less than 0.30%. The maximum difference in D2cm^3^ for OARs was 2.64%. Weishaupt et al. (32) successfully automated the digitization of titanium applicator in formatted CT images of prostate cancer patients undergoing HDR-BT. They first employed a U-Net architecture for automatic segmentation of the applicator contours in 2D sagittal plane slices and then applied a density-based clustering algorithm to classify the 3D applicator. The results demonstrated excellent consistency, with deviations in the apical and axial directions of -0.1 ± 0.6 mm and 0.13 ± 0.09 mm, respectively, compared to the standard manual reconstruction. Xie et al. (33) conducted training and testing based on a dataset of 70 cervical cancer patients who underwent CT-guided HDR-BT treatment, utilizing the nnU-Net network. The average DSC for automatic segmentation of three interstitial metal needles were 0.88, 0.89, and 0.90, respectively, and there were no significant differences in organ structure dose volume metrics between the manual and automatic reconstruction methods. Wang et al. (34) proposed an attention-gated AI model for the automatic digitization of metal interstitial needles in CT-guided cervical cancer HDR-BT. The results demonstrated that a 3D convolutional neural network (CNN) with spatial and channel attention mechanisms outperformed traditional CNNs in needle feature extraction. This method significantly improved the accuracy of interstitial needle localization, reducing the positioning errors of the needle tip and needle axis to 1.1 mm and 1.8 mm, respectively, from 2.0 mm and 3.3 mm. For MRI-guided HDR-BT, Dai et al. (35) developed a U-Net model incorporating an attention-gating mechanism and employed total variation regularization to enhance the accuracy of applicator localization. In the detection of 299 source catheters from 20 patients, the localization errors of the applicator tip and axis were 0.37 ± 1.68 mm and 0.93 ± 0.50 mm, respectively, demonstrating excellent accuracy and reliability. Shaaer et al. (36) utilized two independent U-net models to automatically segment plastic applicator in T1-weighted (T1W) and T2-weighted (T2W) MRI images, respectively. Subsequently, by integrating the catheter location information from the T1W and T2W images through a post-processing step, the complementary information from the two sequences improved the accuracy of the automatic reconstruction. The results exhibited remarkable accuracy, with an average deviation of only 0.97 ± 0.66 mm for automatic reconstruction relative to the standard manual reconstruction.

AI technology significantly reduces the time required for applicator reconstruction in HDR-BT and enhances reconstruction accuracy and consistency. However, strict quality control procedures must be implemented before applying an automatic applicator reconstruction model in clinical practice, with particular attention to factors such as image quality and artifacts that may impact the detection of the source applicator tip. These factors must be comprehensively considered during the model development and performance evaluation stages to ensure the model’s stability and accuracy.

### Plan optimization

Plan optimization is a crucial component of the HDR-BT treatment planning and includes both forward and inverse optimization methods. Early HDR-BT plans often employed forward optimization, where medical physicists manually adjusted the dwell positions and durations of the radiation source to meet clinical dose targets. This approach is heavily dependent on the experience of medical physicists and is a time-intensive process. As a result, inverse optimization techniques, which offer greater efficiency, have gained wider adoption in clinical settings. Inverse optimization calculates variables such as dwell positions and times by establishing objectives and constraints based on clinical requirements for the target area and OARs. This ensures that the desired clinical requirement is achieved in the target area while adhering to dose limitations for OARs. Currently, two inverse optimization algorithms, inverse planning simulated annealing (IPSA) (37) and hybrid inverse treatment planning optimization (HIPO) (38), are commonly applied in commercial treatment planning systems. Several studies have successfully utilized deep reinforcement learning (DRL) to optimize HDR-BT plans for cervical cancer. Shen et al. (39) developed a weight-tuning policy network (WTPN) that optimizes weight factors for various organs based on the dose volume histogram of the plan. Their results indicated that the WTPN generated plans with a quality score approximately 8.5% higher than that of initial plans and 10.7% higher than manually designed plans by medical physicists. Pu et al. (40) introduced an intelligent treatment planning network (ITPN) based on DRL, which enhances treatment plan quality by adjusting dwell times of the radiation source. Compared to the traditional IPSA optimization method, ITPN showed superior performance in improving dose distribution, homogeneity, and conformity index for the bladder, rectum, and sigmoid colon. Kallis et al. (41) optimized dwell times using 3D dose prediction, demonstrating the potential for improved efficiency and consistency in treatment planning. Oud et al. (42) proposed a multi-criterial automatic planning method, achieving comparable or better target dose distribution while reducing exposure to the bladder and rectum. The work of Dickhoff et al. (43) introduced an adaptive objective configuration method, utilizing the multi-objective real-valued gene-pool optimal mixing evolutionary algorithm (MO-RV-GOMEA) to balance target coverage and organ protection for individual patients, addressing complexity and personalized needs. Stenhouse et al. (44) validated the effectiveness of a machine learning (ML) model in selecting intracavitary tubes and hybrid interstitial needles, finding that replanning with ML-predicted configurations improved plan quality and reduced radiation exposure to OARs.

AI has shown great promise in optimizing HDR-BT plans for cervical cancer, enhancing work efficiency, plan homogeneity, and overall quality, while reducing potential errors associated with manual planning. However, it is important to acknowledge that the current body of research primarily focuses on single-tube or specific types of applicators, which may limit the generalizability of AI models to broader contexts.

### Dose calculations

Dose calculations in high-dose-rate brachytherapy (HDR-BT) are typically based on the dose distribution model outlined in the TG-43 report. This model characterizes the behavior of radiation emitted from a radioactive source in water, taking into account the source’s radioactive activity, dwell position, and dwell time. However, it does not consider the potential effects of applicator attenuation and tissue heterogeneity (45). The Monte Carlo (MC) algorithm, a gold standard in radiotherapy dose calculations, determines dose deposition by statistically modeling the probability distribution of particles within the human body (46). However, the lengthy computation times associated with MC algorithms limit their applicability in time-sensitive HDR-BT workflows. To address this, Mao et al. (47) developed RapidBrachyDL, a 3D deep neural network (DNN) model capable of rapidly predicting radiation dose distribution for ^192^Ir-based HDR-BT. The model exhibited high consistency with the MC method regarding dose volume histogram (DVH) and critical dose metrics, coupled with a 300-fold enhancement in computation speed. Additionally, Akhavanallaf et al. (48) created an MC-based personalized dose simulator (PBrDoseSim) and employed DNN to predict patient-specific dose distributions. Their results demonstrated a strong agreement between the DNN-predicted dose and the MC method, with a computational efficiency improvement of approximately 5,400 times compared to conventional MC techniques. Generative adversarial networks (GANs) have emerged as a powerful tool for optimizing dose distribution in HDR-BT. Oud et al. demonstrated the use of GANs for personalized treatment planning, achieving multi-objective optimization that balances target coverage and organ protection. This approach enhances the precision and efficiency of dose calculations, paving the way for more personalized and effective HDR-BT treatment plans (42).

While the aforementioned studies highlight the potential of AI in HDR-BT dose calculations, current research in this domain remains limited, and existing studies lack adequate clinical validation. Moreover, although AI methods significantly enhance computational efficiency, they necessitate substantial computational resources for model training and implementation, which may strain the computational capabilities of certain medical institutions.

## Conclusion

This paper presents a summary of the recent developments in the application of AI technology for cervical cancer high-dose-rate brachytherapy (HDR-BT) treatment planning. AI has demonstrated significant potential in the automatic segmentation of target areas and OARs, applicator reconstruction, plan optimization, and dose calculation. With the help of AI technology, the process of cervical cancer HDR-BT treatment planning is expected to be streamlined by achieving full automation and standardization, thus reducing treatment preparation time, alleviating patient discomfort, and enhancing treatment accuracy and efficacy. However, it is important to acknowledge that the application of AI in this field remains in its initial exploratory stages and faces several challenges, including limited high-quality training data, intricate model construction and optimization processes, and suboptimal result repeatability. To address these challenges and advance the field, future endeavors should focus on the development of open-source algorithm frameworks and the availability of large-scale open datasets to enhance research reproducibility and improve the generalizability and credibility of AI models. Additionally, establishing standardized data collection and annotation procedures, as well as enhancing the interpretability of models, are crucial directions for future research to facilitate the effective translation of AI technology into clinical practice.
